# Micronutrient supplementation adherence and influence on the prevalences of anemia and iron, zinc and vitamin A deficiencies in preemies with a corrected age of six months

**DOI:** 10.6061/clinics/2016(08)06

**Published:** 2016-08

**Authors:** Brunnella Alcantara Chagas de Freitas, Luciana Moreira Lima, Maria Elisabeth Lopes Moreira, Silvia Eloiza Priore, Bruno David Henriques, Carla Fernanda Lisboa Valente Carlos, Jusceli Souza Nogueira Sabino, Sylvia do Carmo Castro Franceschini

**Affiliations:** I Universidade Federal de Viçosa (UFV), Departamento de Medicina e Enfermagem, Viçosa/MG, Brazil; II Fundação Oswaldo Cruz, Instituto Fernandes Figueira, Rio de Janeiro/RJ, Brazil; III Universidade Federal de Vicosa (UFV), Departamento de Nutrição e Saúde, Viçosa/MG, Brazil; IV Centro Viva Vida de Referência Viçosa e Região de Saúde, Viçosa/MG, Brazil

**Keywords:** Infant, Premature, Anemia, Iron Deficiency, Zinc Deficiency, Medication Adherence, Vitamin A, Micronutrients

## Abstract

**OBJECTIVE:**

To analyze adherence to the recommended iron, zinc and multivitamin supplementation guidelines for preemies, the factors associated with this adherence, and the influence of adherence on the occurrence of anemia and iron, zinc and vitamin A deficiencies.

**METHODS:**

This prospective cohort study followed 58 preemies born in 2014 until they reached six months corrected age. The preemies were followed at a referral secondary health service and represented 63.7% of the preterm infants born that year. Outcomes of interest included high or low adherence to iron, zinc and multivitamin supplementation guidelines; prevalence of anemia; and prevalences of iron, zinc, and vitamin A deficiencies. The prevalence ratios were calculated by Poisson regression.

**RESULTS:**

Thirty-eight (65.5%) preemies presented high adherence to micronutrient supplementation guidelines. At six months of corrected age, no preemie had vitamin A deficiency. The prevalences of anemia, iron deficiency and zinc deficiency were higher in the low-adherence group but also concerning in the high-adherence group. Preemies with low adherence to micronutrient supplementation guidelines were 2.5 times more likely to develop anemia and 3.1 times more likely to develop zinc deficiency. Low maternal education level increased the likelihood of nonadherence to all three supplements by 2.2 times.

**CONCLUSIONS:**

Low maternal education level was independently associated with low adherence to iron, zinc and vitamin A supplementation guidelines in preemies, which impacted the prevalences of anemia and iron and zinc deficiencies at six months of corrected age.

## INTRODUCTION

Micronutrient deficiencies, which appear in a context, are not only determined by biological factors but also by socioeconomic and cultural conditions 1,2.

Iron, an essential nutrient, is involved in many vital metabolic processes 3. Iron deficiency is simultaneously the most frequent and most neglected nutritional deficiency and it is the main cause of childhood anemia 4. Zinc is needed for all enzymatic processes associated with intermediate metabolism, from cell growth and differentiation to protein, carbohydrate and lipid metabolism. Iron deficiency is estimated to affect one-third of the global population and ranks fifth among the risk factors for diseases in developing countries 5. Essential for adequate growth and development during childhood, vitamin A participates in epithelial differentiation and maintenance. Its deficiency is concerning for groups at low socioeconomic levels 6.

Preemies are at greater risk of developing iron, zinc and vitamin A deficiencies because they have low stores of these nutrients 7 and because they are suddenly exposed to an environment that exacerbates existing nutritional deficiencies after umbilical cord clamping 6. Their vulnerability is also increased by low successful breastfeeding rates 8, unknown nutritional requirements 9, and quantitatively and qualitatively inadequate monitoring by their families and health care teams at the various levels of care 10.

The Brazilian Society of Pediatrics 11 has provided guidelines for iron, zinc and multivitamin supplementation for preemies. However, the adherence rates to these guidelines and the factors that influence them are unknown. Even when supplements are provided for free and mothers are instructed on their use, mothers often do not give the correct dosage or use the supplements for the correct length of time, which may be influenced by information accuracy regarding the use of the medication and by a culture of using medications for treatment as opposed to prophylaxis 12,13. Thus, monitoring of adherence by healthcare professionals is a must 13, as adherence is a dynamic process of shared responsibility between a patient and their health team 14.

The present study aimed to analyze adherence to the recommended iron, zinc and multivitamin supplementation guidelines for preemies; the factors associated with this adherence; and the influence of adherence on the prevalence of low serum levels of iron, zinc and vitamin A that are characteristic of anemia and other deficiencies.

## METHODS

This prospective cohort study included 58 preemies born at the Hospital São Sebastião (HSS) in the municipality of Viçosa-MG from January 01, 2014, to December 31, 2014. They were followed at the Centro Integrado Viva Vida de Referência Secundária da Microrregião Viçosa (CVV) until reaching six months of corrected age for preemies (CAP).

### Population characteristics and study location

The HSS, where all children from the microregion of Viçosa are born, has served as a referral hospital for high-risk pregnancies since 2009. It has had a human milk bank since 2005 and a neonatal intensive care unit (NICU) since 2004.

The CVV focuses on mother-child health. It is the only referral service for the treatment of preemies in the microregion of Viçosa, which includes 20 small municipalities and encompasses a population of approximately 227,203 inhabitants. Prematurity rates in the microregion vary from 8.8 to 9.9%. The preemies are followed by a multidisciplinary team consisting of pediatricians, nurses, dietitians, psychologists, physiotherapists and social assistants. The CVV works with the Federal University of Viçosa. All preemies discharged from the HSS are sent to the CVV for growth and development monitoring.

### Inclusion and exclusion criteria

The following inclusion criteria were applied: premature birth, born at the HSS, followed at the CVV and legal guardian agreement to study participation. The following exclusion criteria were applied: genetic syndromes, severe congenital malformations, laboratory-confirmed congenital infections, severe chronic diseases, thalassemia, sickle cell trait, legal guardians who refused to join the study or chose to drop out at any time during the study based on the ethical principles of the study, three failed contact attempts, missing three or more appointments and a highly-sensitive C-reactive protein (hs-PCR) test result above 5 mg/L.

### Study variables

The following outcome variables were collected at six months of CAP: high adherence or low adherence to iron, zinc and multivitamin supplementation guidelines; anemia; iron deficiency; zinc deficiency; and vitamin A deficiency. Explanatory variables were analyzed to determine their associations with the outcomes.

Gestational age (GA) was defined as the best estimate between early gestational ultrasound, date of the last period, obstetrical notes and clinical examination using the New Ballard Score. Chronological age (CA) was defined as the postnatal age and the CAP was defined as the GA at birth plus the postnatal age 15.

Iron, zinc, and a multivitamin were supplemented as recommended by the Brazilian Society of Pediatrics 11, adjusting the daily doses according to the preemie’s body weight at the last monthly visit. CA at the introduction of iron, zinc and multivitamin supplementation; adherence to supplementation guidelines; and intolerance to each of the supplements were recorded.

Iron supplementation began at 30 days of life (CA) or when the preemie attended the first outpatient visit, but not sooner than 15 days after the last blood transfusion 16. The oral iron supplement used should be given one hour before the feeding corresponding to lunch. The daily iron dose was based on birth weight (BW): (a) 2 mg/kg/day for preemies with a BW >1500 g; (b) 3 mg/kg/day for preemies with a BW between 1000 and 1500 g; and (c) 4 mg/kg/day for preemies with a BW <1000 g 11.

Multivitamin supplementation was started at 10 days of life (CA) or when the preemie attended the first outpatient visit. A recommended dose of 12 drops a day given orally in the afternoon was used for vitamin A and vitamin D 11.

Zinc was supplemented with an oral dose of 0.5 mg/kg/day of zinc sulfate from 36 weeks (GA) (or when the preemie attended the first outpatient visit) to 6 months of CAP. A solution containing 10 mg/mL of zinc sulfate was given in the early morning 11.

Supplementation adherence was investigated at all visits by interview and verification of supplement volumes inside of flasks. The World Health Organization (WHO) 17 defines adherence as the extent to which a person’s behavior corresponds with agreed recommendations from a health care provider; in this case, adherence was represented by taking the supplements as directed. However, there is no consensus on what ingested percentage constitutes adequate adherence 14. At the beginning of the study and throughout the study course, the mothers received flasks of each supplement and instructions for their use. High adherence was defined as ingestion of at least 75% of the recommended amount and low adherence was defined as ingestion of less than 75% of the recommended amount 13,18. If the supplement volume could not be checked, high adherence was defined as supplementation for at least 75% of the recommended period 13,18. Adequate micronutrient adherence was defined as high adherence to all three supplements by at least 75% of the preemies. Inadequate micronutrient adherence was defined as high adherence in fewer than 75% of the preemies.

Supplement intolerance was characterized by gastrointestinal effects, such as abdominal discomfort, constipation, nausea, vomiting and diarrhea.

Blood was collected on two occasions, at the first month of CAP (first outpatient visit) and at six months of CAP. The following tests were conducted: full blood count, hs-CRP, ferritin, serum zinc, and serum retinol. Five milliliters of preemie venous blood were collected in Vacutainer® tubes, after which 3 mL were mixed with EDTA and 2 mL remained free of anticoagulant 13.

Blood collection was postponed for two weeks if the preemie had any of the following symptoms two weeks before the collection: fever, diarrhea, trivial acute infection, hospitalization, respiratory intercurrence, or other diseases 4. Because infection and inflammation affect ferritin, serum retinol and serum zinc levels, hs-CRP was used to detect these conditions to eliminate confounders 4. A new blood sample was collected two weeks later from all preemies with hs-CRP higher than 5 mg/L 4. If a new blood collection was not possible, the test result was not included in the analysis.

Anemia at six months of CAP was defined as hemoglobin (Hb) below 10.0 g/dL 19-21. However, between weeks six and eight, lower normal cutoffs were used, namely, 8.5 g/dL (<1500 g) and 9.0 g/dL (1500 g-2000 g); and at ten weeks, cutoffs of 9.0 g/dL (<1500 g) and 9.5 (1500 g-2000 g) were used 3,19,22. Children over six months of CAP were considered anemic when their Hb was below 11.0 g/dL 1.

The minimum normal ferritin level was defined as 30 ng/mL 23. Vitamin A deficiency was defined as serum retinol below 0.2 mg/L.^4^ Zinc deficiency was defined as serum zinc below 70 µg/dL 24.

The collected sociodemographic variables were maternal and paternal age, maternal and paternal education level (fewer or more than eight years of formal education), maternal marital status (single/divorced, married/has partner), family income in minimum salaries (MS; <2 MS and ≥2 MS) 25, income per capita (MS), and number of household dwellers. The minimum salary in 2014 was R$ 724.00.

The collected prenatal and perinatal variables were type of delivery, sex, GA (weeks), birth weight (BW), stratification by BW and GA, BW adequacy for GA, NICU stay (yes/no) and duration of NICU stay. BW was categorized as <1000 g, 1000-1499 g, 1500-2499 g, and ≥2500 g and GA as <28 weeks, 28 to <32 weeks, and ≥32 weeks 15. Fenton curves 26 were used to classify the preemies as small for gestational age (SGA < tenth percentile), adequate for gestational age (tenth percentile < AGA < ninetieth percentile), and large for gestational age (LGA > ninetieth percentile).

Feeding at the first visit was categorized as exclusive breastfeeding (EBF), complementary feeding (CF) and formula feeding (FF) 27. Total breastfeeding duration was recorded, taking into account the CAP in months. Inappropriate diets were also recorded. These included cow milk, yogurt, incorrect formula dilution, incorrect composition of the main soft food, addition of cereals to milk, and use of teas.

### Power and sample size

Based on a hypothesis test of a proportion, an estimated high adherence rate to micronutrient supplementation of 75% (because the preemies were followed by a multidisciplinary team at a referral secondary service), a literature-based high adherence rate of 56.7% (for the municipality of Viçosa 13)), a significance level of 5% and a power of 85%, the sample was determined to require at least 48 preemies. The study included 58 preemies.

### Statistical analysis

For descriptive analysis, quantitative variables were expressed as the mean, standard deviation, median, 25th percentile and 75th percentile. Qualitative variables were expressed as absolute values and percentages. The Kolmogorov-Smirnov test was used to determine whether the quantitative variables had a normal distribution. Variables with a parametric distribution were expressed as the mean and standard deviation and those with a nonparametric distribution were expressed as the median with the 25^th^ and 75^th^ percentiles. The frequencies were based on the total number of valid answers. Missing data were not included.

The outcomes of high adherence and low adherence were associated with the variables anemia, iron deficiency, zinc deficiency, vitamin A deficiency, the laboratory values that characterized them and the other explanatory variables.

Pearson’s chi-square test or Fisher’s exact test (when necessary) was used to compare the categorical variables. Student’s t-test or the Mann-Whitney test was used to compare the means or medians of the quantitative variables.

The measures of effect were the prevalence ratios (PRs) provided by Poisson regression. Initially, each covariate was submitted to univariate analysis for the event of interest and the final multiple regression model included the explanatory variables with *p* values <0.20 in the univariate analysis to control for possible confounding factors. The Poisson multiple regression model was analyzed by robust estimators of variance to avoid overestimates of the PRs and its adjustment was verified by the goodness-of-fit test 28. The final model included the variables with a significance level of 5% (*p*<0.05).

The statistical analyses were performed using Statistical Package for the Social Sciences (SPSS), version 20.0 and Stata 9.0.

### Ethical aspects

Only preemies whose legal guardians agreed to participate in the study by signing an informed consent form were included. This study was conducted in compliance with the norms and guidelines for human research provided by Resolution no. 466 passed on December 12, 2012, by the National Health Council of the Department of Health, Brasilia, DF. The study also complies with the 1975 Declaration of Helsinki, revised in 1983.

The study was approved by the Human Research Ethics Committee of UFV under protocol number 675,427/2014 and is associated with a doctoral thesis developed in the graduate program in nutrition of the Department of Nutrition and Health of UFV.

## RESULTS

Ninety-one preemies were born during the study period, and 66 (72.5%) attended the CVV. Two preemies were not included in the study, one because the parents did not agree to participate and another because of severe congenital malformation. Six preemies dropped out, representing a loss to follow-up of 12.1%. Hence, 58 preemies participated in the study, representing 63.7% of the preemies born that year. The sociodemographic and perinatal characteristics of the group that remained in the study and the dropouts did not differ (data not shown).

Tables 1 and 2 show the sociodemographic and perinatal characteristics of the preemies and their families. In the first outpatient visit, the mean CAP was 41.7±3.0 weeks. None of the preemies were taking zinc at the first outpatient visit, 35.3% were taking iron, and 42.2% were taking a multivitamin. The adherence rate for all three supplements (iron, zinc and multivitamin) was 65.5%. The adverse events associated with ferrous sulfate included nausea, vomiting, abdominal discomfort and constipation. These events affected 18.8% of the preemies but resolved once ferrous sulfate was replaced by other compounds (iron(III)-hydroxide polymaltose complex (IPC), iron chelate, or iron bis-glycinate chelate). The multivitamin caused nausea and vomiting in 6.9% of the preemies, but these symptoms resolved when another multivitamin was used. Zinc supplementation did not cause any adverse events. The doses of the new supplements were not changed. Table 3 shows these data.

At the first month of CAP, the mean and median laboratory values of the preemies with high and low supplement adherence did not differ (Table 4). However, at six months of CAP, the mean and median red blood cell count (except for anisocytosis, which presented an inverse behavior) and ferritin and zinc levels were significantly lower in the low-adherence group (Table 5).

At one month of CAP, the prevalences of anemia and iron, zinc and vitamin A deficiencies were 36.7%, 25.7%, 33.3% and 24.4%, respectively. At six months of CAP, the prevalences of anemia, iron deficiency and zinc deficiency were 38.3%, 68.9% and 36.4%, respectively. All preemies had normal vitamin A levels.

At six months of CAP, the prevalences of anemia (64.3%) and zinc deficiency (75%) were significantly higher in the group with low adherence and all preemies in this group presented with iron deficiency. According to bivariate Poisson regression, low adherence to the supplements increased the likelihood of anemia by 2.5 times (PR 2.52; 95%CI 1.04-6.07; *p*=0.04) and the likelihood of zinc deficiency by 3.1 times (PR 3.12; 95%CI 1.25-7.74; *p*=0.014). These data were not tabulated.

Even in the group with high supplement adherence, the prevalences of anemia (27.3%), iron deficiency (57.6%) and zinc deficiency (21.9%) were concerning.

The explanatory variables were submitted to univariate analysis for the outcomes high and low adherence. The variables with a *p* value <0.20 were included in the final multiple regression model – Poisson regression with robust estimators of variance – and its adjustment was verified by the goodness-of-fit test (Table 6). In the final model, low maternal education level increased the likelihood of low adherence to the three supplements by 2.2 times (PR 2.23; 95%CI 1.01-4.93; *p*=0.047).

## DISCUSSION

Some study preemies had inadequate adherence to the iron, zinc, and multivitamin supplement guidelines recommended by the Brazilian Society of Pediatrics 11 because the expected adherence rate should exceed 75%. Inadequate adherence occurred regardless of the facts that in the first six months of CAP, the preemies were followed by a multidisciplinary team at a referral secondary service, the supplements were provided free of charge and in all visits, the families were informed of the need for supplementation and taught how to administer the supplements and adherence was checked.

Although zinc supplementation should start no sooner than week 36 of CAP, none of the preemies were taking zinc by week 41 of CAP, when the first outpatient visit occurred. Conversely, 35.3% of the preemies who attended the first visit within at least 30 days of life were taking the iron supplement and 42.2% were taking the multivitamin. Supplementation, especially of zinc, was neglected or forgotten by the study mothers and health professionals, which has been reported elsewhere 13.

In a recent meta-analysis, Jin et al. 29 compared the effects of early iron supplementation (introduced with enteral feeding before three weeks of life) and late iron supplementation (introduced between ages four weeks and 60 days) and concluded that early iron supplementation is associated with a lower decrease in ferritin and Hb levels in preemies. However, the study also warned about the need to monitor iron levels to avoid iron overload and other possible negative effects.

Micronutrient deficiencies originate within a wide context, and their occurrence is determined not only by biological factors but also by socioeconomic and cultural conditions 1,2. Even when supplements are provided free of charge and mothers receive instructions from a health care team, they often do not administer supplements to their children at the correct dosages or for the correct length of time 12. This phenomenon was reported in a study conducted with breastfed infants from Viçosa 13, where only 56.7% of the infants presented high adherence to prophylactic iron supplementation. In this same study, low adherence was attributed to lack of knowledge regarding the use of ferrous sulfate to prevent anemia and a culture of using medications for treating as opposed to preventing disease 13.

The preemies examined in the present study already presented micronutrient deficiencies at the beginning of the outpatient follow-up, at the first month of CAP. The prevalences of anemia, iron deficiency, zinc deficiency, and vitamin A deficiency were 36.7%, 25.7%, 33.3%, and 24.4%, respectively. In the beginning of the study, the prevalences of micronutrient deficiencies in the two groups of preemies, who were later categorized as high or low adherence, did not differ. Micronutrient stores in preemies are rapidly depleted in the first weeks after birth, and their fast growth rate is associated with higher micronutrient requirements 7,30,31. Yamada et al. 32 compared late preemies with term infants and found lower Hb levels and iron stores in preemies one month after birth, which reinforces the need for iron supplementation in this population. Fares et al. 33 found a high incidence of vitamin A deficiency (75.9%) in very low birth weight preemies. During the first month of life, preemies’ fat absorption mechanisms are not yet fully developed 34. Additionally, there is evidence of an association between maternal micronutrient deficiencies and their occurrence in preemies 35,36.

At the end of the study, at six months of CAP, the prevalences of anemia, iron deficiency, and zinc deficiency were 38.3%, 68.9%, and 34.6%. In a Brazilian sample of very low birth weight preemies followed at a referral health service, Ferri et al. 37 found rates of anemia and iron deficiency of 26.5% and 48%, respectively. There are well established reference values for Hb 1, allowing for comparisons of studies. However, the highest rates of iron deficiency found in the present sample can be attributed to the different cut-off points used 23.

Zinc deficiency has been described in breastfed preemies under 34 weeks of GA and may be explained by the relative inability of breast milk to supply the zinc required by a preemie. Thus, proper follow-up and supplementation of children at risk of micronutrient deficiencies, such as preemies, are necessary 7,30. In addition to the high prevalences of iron and zinc deficiencies in developing countries, preemies have low micronutrient stores, so they are at risk of developing deficiencies after birth if they do not receive supplementation 1,7.

The preemies evaluated in the current study with low supplement adherence were 2.5 and 3.1 times more likely to develop anemia and zinc deficiency, respectively. Iron deficiency was not included in the bivariate Poisson regression because one of the slots had a frequency of zero, but all the preemies with low adherence had iron deficiency.

The preemies in the current study who experienced adverse gastrointestinal events secondary to the use of ferrous sulfate and a multivitamin received different iron and multivitamin formulations, which resolved the problem. The study results indicate that health professionals must prescribe the recommended micronutrients and follow patient adherence.

Even in the high-adherence group, the prevalences of anemia, iron deficiency and zinc deficiency were concerning. Known risk factors for anemia and weaning are early introduction of cow milk and introduction of low-iron complementary foods 37,38. The present study did not intend to analyze the factors associated with anemia and micronutrient deficiencies, but in the first outpatient visit, 81% of the preemies were breastfeeding. By the end of the study, 44.7% of those preemies were on formula and 53.4% of the preemies were on inappropriate diets. Given these observations, future studies should analyze the risk factors for anemia and iron and zinc deficiencies and review the recommended micronutrient doses.

Low maternal education level was associated with a 4.5-fold greater likelihood of low supplement adherence. Greater vulnerability was found in preemies whose families had low per capita income and low education level. Low socioeconomic and education levels have already been correlated with low supplement adherence elsewhere. Moreover, micronutrient deficiencies are more prevalent in groups of lower socioeconomic status because they have greater risk factor exposure 10.

Adherence is compromised when parents do not understand the impact of a disease or the importance of a medication 13. Although all of the studied preemies had the same access to multidisciplinary care and received supplements free of charge, only low maternal education level was associated with low supplement adherence, regardless of other sociodemographic and perinatal characteristics.

Health professionals need to develop strategies to provide health education to mothers of low education level to increase preemie adherence to iron, zinc and multivitamin supplement guidelines 11. Health professional training, cooperation and motivation are factors that can exert a positive influence on mothers with low education level, who play a critical role in adherence to the micronutrient supplementation recommendations prescribed for their preemies 11,14. Preemies need to be monitored and strategies must be assessed to improve intervention effectiveness 13,18.

The study limitations include the small sample size, which hindered analyses of incidence and risk and only allowed assessments of prevalences, despite the adequate power. However, the wide confidence intervals observed in the univariate analysis were corrected using Poisson regression with robust estimators of variance.

The study strengths include the longitudinal design, which allowed the observation of preemies over a period of time and enabled determination of how micronutrient supplementation adherence influenced the prevalences of deficiencies; assessment of adherence to the micronutrient regime recommended by the Brazilian Society of Pediatrics; and assessment of the impact of supplement adherence on the prevalences of anemia and iron and zinc deficiencies. The present study aims to serve as an incentive and foundation for other studies on this topic.

Of all the study parameters, the most important finding was that low maternal education level was independently associated with low adherence to iron, zinc and multivitamin supplementation guidelines in preemies, which affected the prevalences of anemia and iron and zinc deficiencies at six months of corrected age for preemies. Health professionals should establish strategies to improve supplement prescription and adherence in this vulnerable population.

This study was conducted for the graduate program in nutrition science of the Federal University of Viçosa - UFV - Viçosa (MG), Brazil.

### Academic affiliations

This article is part of the doctoral thesis of Brunnella Alcantara Chagas de Freitas from the Federal University of Viçosa – UFV.

## AUTHOR CONTRIBUTIONS

Freitas BA collected and analyzed data and wrote the manuscript. Carlos CF collected data and wrote the manuscript. Sabino JS collected data. Lima LM, Moreira ME, Priore SE, Henriques BD and Franceschini SC supervised the analyses and reviewed the manuscript.

## Figures and Tables

**Table 1:** Perinatal characteristics of the preemies and their mothers. Viçosa-MG, 2014 (n=58).

Variables	n (%)	Mean (±SD)	Median (P25-P75)
Sex			
Female	24 (41.4)	--	--
Male	34 (58.6)		
GA (weeks)	58 (100.0)	--	34.8 (32.0-35.7)
Birth weight (g)	58 (100.0)	1998.6 (563.8)	--
GA			
<28 weeks	3 (5.2)	--	--
≥28 and <32 weeks	8 (13.8)		
≥32 weeks	47 (81.0)		
Birth weight			
<1000 g	3 (5.2)	--	--
1000-1499 g	9 (15.5)		
1500-2499 g	34 (58.6)		
≥ 2500 g	12 (20.7)		
BW/GA ratio*			
AGA	43 (74.1)	--	--
SGA	12 (20.7)		
LGA	3 (5.2)		
Twins			
Yes	14 (24.1)	--	--
No	44 (75.9)		
NICU stay			
Yes	33 (57.9)	--	--
No	24 (42.1)		
Length of stay (days)	33 (56.9)	29.2 (27.8)	--
Red blood cell transfusion			
Yes	10 (17.5)	--	--
No	47 (82.5)		
Type of delivery			
Caesarian	40 (69.0)	--	--
Vaginal	18 (31.0)		
Parity	--	--	1.0 (1.0-2.0)
Number of prenatal visits	--	6.9 (2.2)	--

The values refer to the total number of valid answers. Missing data were not included. SD: standard deviation; GA: gestational age; BW: birth weight; AGA: adequate for gestational age; SGA: small for gestational age; LGA: large for gestational age; NICU: neonatal intensive care unit

* Fenton curves 2013(26).

**Table 2:** Sociodemographic characteristics of the preemies and their families. Viçosa-MG, 2014 (n=58).

Variables	n (%)	Mean (±SD)	Median (P50-P75)
Family income (in MS)*	54 (91.5)	--	1.8 (1.0 - 2.0)
Per capita income (in MS)*	54 (91.5)	--	0.4 (0.2 – 0.6)
Individuals per household	56 (94.9)	--	3.0 (2.0 - 4.0)
Maternal age	58 (100.0)	25.5 (6.7)	--
Paternal age	58 (100.0)	27.8 (6.6)	--
Maternal marital status			
Married/has partner	44 (81.5)	--	--
Single/divorced	10 (18.5)		
Maternal education level			
≤ 8 years	24 (42.1)	--	--
> 8 years	33 (57.9)		
Paternal education level			
≤ 8 years	33 (60.0)	--	--
> 8 years	22 (40.0)		

The values refer to the total number of valid answers. Missing data were not included. SD: standard deviation; MS: minimum salary (in 2014 = R$ 724.00).

**Table 3:** Characterization of diet and iron, zinc, and multivitamin supplementation for the preemies during their first six months of corrected age. Viçosa-MG, 2014 (n=58).

Variables	n (%)	Mean (±SD)	Median (P25-P75)
Diet at the first visit		--	--
EBF	22 (37.9)		
CF	25 (43.1)		
FF	11 (19.0)		
Breastfed at the end of the study		--	--
Yes	26 (55.3)		
No	21 (44.7)		
Inappropriate diet			
Yes	31 (53.4)	--	--
No	27 (46.6)		
Was taking the following at the first visit			
Iron*			
Yes	18 (35.3)	--	--
No	33 (64.7)		
Multivitamin**			
Yes	27 (42.2)		
No	37 (57.8)		
Zinc***			
Yes	--		
No	58 (100.0)		
Initial age			
Iron (CA)	58 (100.0)	--	1.1 (1.0-1.6)
Multivitamin (CA)	58 (100.0)	--	1.0 (0.3-1.2)
Zinc (CAP)	58 (100.0)	44.0 (7.0)	--
Adherence to iron salts		--	--
High	40 (69.0)		
Low	18 (30.0)		
Adherence to multivitamin		--	--
High	39 (67.2)		
Low	19 (32.8)		
Adherence to zinc		--	--
High	40 (69.0)		
Low	18 (31.0)		
Adherence to all three supplements		--	--
High	38 (65.5)		
Low	20 (34.5)		
Intolerance to ferrous sulfate****		--	--
Yes	9 (18.8)		
No	39 (81.2)		
Intolerance to the multivitamin		--	--
Yes	4 (6.9)		
No	54 (93.1)		

The values refer to the total number of valid answers. Missing data were not included. SD: standard deviation; med: median; EBF: exclusive breastfeeding; CF: complemented feeding; FF: formula feeding; CA: chronological age (months); CAP: corrected age for preemies (weeks). Inappropriate diet included introduction of cow milk, yogurt, incorrect formula dilution, incorrect composition of the main meal (soft food), addition of cereals to milk, and use of teas.

* Includes preemies with a CAP ≥1 month in the first visit (of the five preemies who received red blood cell transfusion less than 15 days before being discharged from the NICU, four were already taking iron salts in the first visit).

** Includes preemies with a CAP ≥7 days in the first visit.

*** Includes preemies with a CAP ≥36 weeks. Preemies who received a dose of supplement lower than the recommended dose were considered not supplemented.

**** Ten preemies were already using another iron compound in the first visit, and this compound was not changed. Therefore, ferrous sulfate intolerance could not be assessed in these preemies. There was no intolerance to zinc.

**Table 4:** Mean and median laboratory values for the preemies at one month of corrected age according to adherence or nonadherence to iron, zinc, and multivitamin supplementation guidelines. Viçosa-MG, 2014.

Variables	High adherence (n=38)			Low adherence (n=20)			*p*-value
	n (%)	Mean (±SD)	Med (min-max)	n (%)	Mean (±SD)	Med (min-max)	
Hb	32 (84.2)	10.6 (1.5)	--	17 (85.0)	11.6 (3.2)	--	0.112*
Htc	32 (84.2)	--	30.6 (26.0-45.1)	17 (85.0)	--	30.2 (24.2-51.9)	0.702**
MCV	32 (84.2)	92.7 (8.5)	--	17 (85.0)	90.2 (13.5)	--	0.438*
MCH	32 (84.2)	30.7 (2.9)	--	17 (85.0)	29.3 (4.9)	--	0.236*
MCHC	32 (84.2)	32.9 (0.6)	--	17 (85.0)	32.2 (1.3)	--	0.088*
RDW	32 (84.2)	12.8 (1.1)	--	17 (85.0)	13.9 (2.1)	--	0.121*
Ferritin	31 (81.6)	--	79.4 (16.9-435.0)	13 (65.0)	--	102.7 (30.9-186.5)	0.827**
Zinc	29 (76.3)	64.6 (14.8)	--	13 (65.0)	65.1 (16.5)	--	0.918*
Vitamin A	30 (78.9)	--	0.2 (0.1-0.3)	13 (65.0)		0.2 (0.1-0.3)	0.442**
Anemia							
Yes	11 (34.4)	--	--	7 (41.2)	--	--	0.648***
No	21 (65.6)	--	--	10 (58.8)	--	--	
Iron deficiency							1.000****
Yes	4 (12.9)	--	--	1 (7.7)	--	--	
No	27 (87.1)	--	--	12 (92.3)	--	--	
Zinc deficiency							0.417****
Yes	5 (16.7)	--	--	9 (75.0)	--	--	
No	25 (83.3)	--	--	3 (25.0)	--	--	

The values refer to the total number of valid answers. Missing data were not included. Cases with high-sensitivity C-reactive protein test results above 5 mg/L were not included. SD: standard deviation; med: median; min: minimum value; max: maximum value; Hb: hemoglobin (g/dL); Htc: hematocrit (%); MCV: mean corpuscular volume (fL); MCH: mean corpuscular Hb (pg); MCHC: mean corpuscular Hb concentration (g/dL); RDW: anisocytosis index (%); ferritin (ng/mL); vitamin A (mg/L); zinc (µg/dL).

* *p*-value according to Student’s t-test.

** *p*-value according to the Mann-Whitney test.

*** *p*-value according to Pearson’s chi-square test.

**** *p*-value according to Fisher’s exact test.

**Table 5:** Mean and median laboratory values for the preemies at six months of corrected age according to adherence or nonadherence to iron, zinc, and multivitamin supplementation guidelines. Viçosa-MG, 2014.

Variables	High adherence (n=38)			Low adherence (n=20)			*p*-value
	n (%)	Mean (±SD)	Med (min-max)	n (%)	Mean (±SD)	Med (min-max)	
Hb	33 (86.8)	11.6 (0.8)	--	14 (70.0)	10.4 (1.1)	--	<0.001*
Htc	33 (86.8)	35.1 (2.3)	--	14 (70.0)	32.3 (2.9)	--	<0.001*
MCV	33 (86.8)	79.1 (3.6)	--	14 (70.0)	72.6 (6.9)	--	0.004*
HCM	33 (86.8)	26.3 (1.4)	--	14 (70.0)	23.5 (3.0)	--	0.005*
CHCM	33 (86.8)	33.1 (0.7)	--	14 (70.0)	32.2 (1.4)	--	0.049*
RDW	33 (86.8)	13.3 (1.1)	--	14 (70.0)	15.1 (2.1)	--	0.010*
Ferritin	33 (86.8)	--	27.3 (3.9-210.2)	12 (60.0)	--	12.6 (4.10-28.3)	0.001**
Zinc	32 (84.2)	84.9 (20.9)	--	12 (60.0)	62.8 (9.2)	--	0.001*
Vitamin A	31 (81.6)	--	0.3 (0.2-0.8)	12 (60.0)	--	0.3 (0.2-0.4)	0.584**
Anemia							0.017***
Yes	9 (27.3)	--	--	9 (64.3)	--	--	
No	24 (72.7)	--	--	5 (35.7)	--	--	
Iron deficiency							****
Yes	19 (57.6)	--	--	12 (100.0)	--	--	
No	14 (42.4)	--	--	--	--	--	
Zinc deficiency							0.003*****
Yes	7 (21.9)	--	--	9 (75.0)	--	--	
No	25 (78.1)	--	--	3 (25.0)	--	--	

The values refer to the total number of valid answers. Missing data were not included. Refers to the cases that adhered to all three supplements. Cases with high-sensitivity C-reactive protein test results above 5 mg/L were not included. SD: standard deviation; med: median; min: minimum value; max: maximum value; Hb: hemoglobin (g/dL); Htc: hematocrit (%); MCV: mean corpuscular volume (fL); MCH: mean corpuscular Hb (pg); MCHC: mean corpuscular Hb concentration (g/dL); RDW: anisocytosis index (%); ferritin (ng/mL); vitamin A (mg/L); zinc (µg/dL).

* *p*-value according to Student’s t-test.

** *p*-value according to the Mann-Whitney test.

*** *p*-value according to Pearson’s chi-square test.

**** The test of association could not be performed because the frequency was equal to zero.

***** *p*-value according to Fisher’s exact test.

**Table 6:** Bivariate and multivariate analyses of the variables included in the model for the outcome adherence to iron, zinc, and multivitamin supplementation guidelines. Viçosa-MG, 2014.

Variables	High adherence (n=38) n (%)	Low adherence (n=20) n (%)	Crude PR (95%CI)*	Adjusted PR (95%CI)**	*p*-value**
Maternal education level					0.047
≤8 years	12 (46.2)	14 (53.8)	4.04 (1.33-12.27)	2.23 (1.01-4.93)	
>8 years	26 (86.7)	4 (13.3)	1.00	1.00	
Paternal education level					--
≤8 years	18 (56.2)	14 (43.8)	9.41 (1.25-70.97)	--	
>8 years	19 (95.0)	1 (5.0)	1.00		
Marital status					--
Single/divorced	4 (40.0)	6 (60.0)	2.29 (0.85-6.19)	--	
Married/has partner	31 (73.8)	11 (26.2)	1.00		
Family income					--
<2MS	15 (55.6)	12 (44.4)	2.00 (0.75-5.33)	--	
≥2MS	21 (77.8)	6 (22.2)	1.00		
Inappropriate diet				--	--
Yes	15 (48.4)	16 (51.6)	3.22 (1.08-9.65)		
No	22 (81.5)	5 (18.5)	1.00		

MS: minimum salary; PR: prevalence ratio; 95%CI: 95% confidence interval.

* Poisson regression.

** Poisson regression with robust estimation of variance.
